# Comparative angiotomographic study of swine vascular anatomy: contributions to research and training models in vascular and endovascular surgery

**DOI:** 10.1590/1677-5449.200086

**Published:** 2021-05-14

**Authors:** Adenauer Marinho de Oliveira Góes, Rosa Helena de Figueiredo Chaves, Ismari Perini Furlaneto, Emanuelle de Matos Rodrigues, Flávia Beatriz Araújo de Albuquerque, Jacob Hindrik Antunes Smit, Carolina Pinheiro de Oliveira, Simone de Campos Vieira Abib

**Affiliations:** 1 Centro Universitário do Estado do Pará – CESUPA, Curso de Medicina, Belém, PA, Brasil; 2 Universidade Federal de São Paulo – UNIFESP, Programa de Ciência Cirúrgica Interdisciplinar, São Paulo, SP, Brasil.

**Keywords:** pigs, blood vessels, vascular surgical procedures, anatomy, comparative, endovascular procedures, computed tomography angiography

## Abstract

**Background:**

Medium and large animal models allow researchers to evaluate the efficacy and safety of cardiovascular procedures in systems that resemble human anatomy and can be used to simulate scenarios for training purposes. Although porcine models have been used extensively, many physiological and anatomical features remain unknown or only superficially described.

**Objectives:**

To describe the normal porcine vascular anatomy on computed tomography scans, compare it to human vascular anatomy, and discuss the application of porcine models for open and endovascular procedures.

**Methods:**

Three male Landrace pigs underwent computed tomography. The vascular anatomy of the neck, thorax, abdomen, and limbs was analyzed and described; relevant similarities and differences between porcine and human vascular anatomies and the implications for vascular procedures in pigs are highlighted.

**Results:**

The carotid territory, aortic arch, and terminal aorta branches all show marked differences in pigs compared to their human counterparts. Compressions of both left renal and common iliac veins were detected, analogous to those seen in human Nutcracker and May-Thurner syndromes. Vascular measurements (diameters, lengths, and angles) of several different porcine territories are presented.

**Conclusions:**

The data presented should be useful for planning preclinical trials and basic research and for refining surgical training using porcine models in vascular fields.

## INTRODUCTION

Cardiovascular diseases are among the most significant health problems and constitute a fundamental target of biomedical research.^1^ Some preliminary studies are conducted with smaller animals (mice, rats, and rabbits), because they offer a good cost-benefit relationship.^2^ Non-human primates (baboons, for example) are also used when there is a need for tests with larger devices, but cost and the complexity of ethical issues are limiting factors. Pig and sheep models constitute important options for research in vascular surgery.^2^^-^ 4 These animals can be used by researchers to assess the efficacy and safety of cardiovascular procedures in systems with similar anatomy to humans and can also be used in training.^1^

Pigs have been used as surgical models for a long time, in particular because they are reasonably priced and have similar cardiovascular anatomy and physiology to human beings.^1^^,^ 3 ^-^ 8 Porcine cardiovascular anatomy and physiology are the most studied, as illustrated by studies on the distribution of coronary arteries, ventricular function, cardiac metabolism, electrophysiology, and development of collateral circulation after acute myocardial infarction.^1^

Recent publication of the genome and the possibility of genetically modifying pigs have increased even further the importance of porcine models in medical research.^4^ One key factor in development of experimental models and in training is knowledge of the anatomy and its variants.^5^ Classic studies, based on surgical dissections, such as “The anatomy of domestic animals”, were published in 1910,^9^ and were followed by others that described use of pigs for a variety of procedures.^1^^,^ 2 ^,^ 5 ^-^ 8 ^,^ 10 ^-^ 16

Over recent decades, the complexity of open and endovascular surgery has been increasing. Noninvasive imaging methods, such as computed tomography (CT), have become routine for treatment planning and monitoring of cases and are progressively substituting traditional angiography. Despite technical developments, there is a lack of detailed descriptions of the extracardiac vascular anatomy of pigs.^3^^,^ 5 ^,^ 6 ^,^ 13

The objective of this study was to describe the characteristics of the vascular anatomy of pigs using CT, compare it to human anatomy, and discuss applications of porcine models for open and endovascular surgical procedures.

## METHODS

This study was approved by the institutional animal research ethics committee.

Three male Landrace pigs were used for CT image acquisition. Their body weights were 45.4 kg, 49.2 kg, and 52.3 kg. The animals were kept in standard conditions (with controlled temperature and humidity) and had fasted for 12 hours before examinations.

### Anesthetic protocol

Procedures were conducted under general anesthesia and monitored by a veterinary physician. Pre-medication comprised intramuscular injection of ketamine (15 mg/kg) and xylazine (1.5 mg/kg).

Anesthesia was induced by intravenous injection of propofol (2.5-5 mg/kg) and maintained with continuous infusion of the same medication (0.1-0.2 mg/kg/min). Animals were positioned in ventral decubitus during acquisition of images. There was no need for intubation.

### Computed tomography

Images were acquired in a 64-channel scanner, with 0.625 mm slices. The intravenous iodinecontrast used was Ioexol (120 mL at a flow velocity of 5 mL/s). Vascular measurements were taken during the arterial phase, using the bolus-tracking technique and the venous phase was initiated immediately after the arterial phase.

After the CT examinations, the animals were used in surgical skills training at the same institution and then euthanized by intravenous injection of potassium chloride.

### Image analysis

Images were viewed on Horos™ v3.1.0 (Horosproject.org) software. For the anatomic descriptions, the terms “ventral” and “dorsal” were adopted as equivalents of “anterior” and “posterior” in humans. Along the same lines, “cranial” and “caudal” were sued as equivalents of “superior” and “inferior”.^17^

The largest diameters of vessels were measured along two perpendicular axes and then the mean diameter in that territory was obtained.

**Figure 1:**
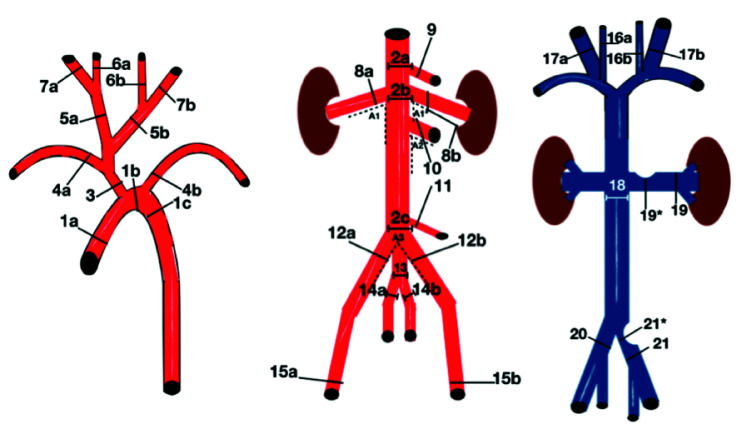
Diagrams illustrating measurement reference points. *:venous diameter measurement at the point of compression.

Measurements were taken at anatomic landmarks related to surgical and endovascular procedures, such as visceral angioplasties, vena cava filter implantation, and aortic procedures (Figure 1).

### Statistical analysis

Means, medians, standard deviations, and coefficients of variation (CV) were calculated using Prism 6 (Graphpad Software, La Jolla, United States).

## RESULTS

### Arterial anatomy

The external carotids had larger diameter than the internal carotids.

The aorta exhibited accentuated and progressive narrowing from its origin to its distal portion, with mean diameter of 25 mm at the ascending aorta and 9.8 mm at the terminal aorta.

The visceral branch with the largest diameter was the cranial mesenteric artery, followed by the celiac artery. The caudal mesenteric artery had the smallest diameter. Arterial diameters are shown in Table 1.

**Table 1:** Diameters arterial.

**Arterial diameters**	**Minimum**	**Maximum**	**Mean**	**SD**	**CV%**
Ascending aorta (1a)	22.9	28.2	25.0	2.8	11.1
Aortic arch (1b)	18.7	24.1	21.5	2.7	12.5
Descending aorta (1c)	16.4	22.5	19.4	3.0	15.6
Abdominal aorta (2a)	12.1	13.4	12.9	0.7	5.4
Abdominal aorta (2b)	8.7	11.1	10.2	1.3	12.6
Abdominal aorta (2c)	9.1	10.8	9.8	0.9	9.1
Brachiocephalic trunk (3)	10.6	13.0	11.5	1.3	11.3
Right subclavian artery (4a)	4.3	6.8	5.8	1.4	23.5
Left subclavian artery (4b)	4.4	6.2	5.6	1.1	19.1
Right common carotid (5a)	4.0	5.6	4.8	0.8	17.0
Left common carotid (5b)	4.7	5.4	4.9	0.4	7.8
Right internal carotid (6a)	3.5	4.0	3.8	0.3	7.7
Left internal carotid (6b)	3.4	3.7	3.5	0.2	5.0
Right external carotid (7a)	4.5	5.5	5.0	0.5	10.6
Left external carotid (7b)	5.1	5.8	5.4	0.4	6.8
Right renal artery (8a)	3.5	5.1	4.4	0.8	18.9
Left renal artery (8b)	5.1	5.7	5.3	0.4	6.7
Celiac trunk (9)	4.8	7.1	6.2	1.2	19.6
Cranial mesenteric artery (10)	6.9	8.2	7.4	0.7	9.8
Caudal mesenteric artery (11)	2.0	2.6	2.4	0.3	13.6
Right external iliac artery (12a)	5.4	8.2	7.2	1.6	22.0
Left external iliac artery (12b)	6.1	7.3	6.7	0.6	8.6
Internal iliac trunk (13)	4.7	8.0	6.1	1.7	28.4
Right internal iliac artery (14a)	3.7	4.7	4.3	0.6	12.7
Left internal iliac artery (14b)	3.8	4.3	4.1	0.3	6, 4
Right superficial femoral artery (15a)	4.9	6.4	5.8	0.8	13.7
Left superficial femoral artery (15b)	4.8	5.6	5.3	0.4	8.2

SD = standard deviation; CV = coefficient of variation; number and letters in parenthesis (for example, 1a) correspond to the measurement points shown on Figure 1.

One of the animals had a left renal artery that was more caudal than the right, while in the other two animals both renal arteries emerged at the same level of the aorta. The angle of the renal arteries in relation to the aorta varied widely, from 82.5º to 114.5º (mean of 96.8 º) on the right and from 76.8º to 116.3º (mean of 93.8º) on the left.

The mean length of the right superficial femoral artery (at the level of the head of the femur) to the terminal aorta (L1) was12.9 cm. Starting from the same point, mean lengths were 22.1 cm to the more caudal renal artery (L2), 30 cm to the celiac artery (L3), and 58.9 cm to the left subclavian artery (L4). Angles and lengths of the arteries are presented in Table 2.

**Table 2:** Arterial angles and lengths.

**Arterial angles/lengths**	**Minimum**	**Maximum**	**Mean**	**SD**	**CV%**
Angle of the right renal artery (A1)	82.5	114.5	96.8	16.3	16.8
Angle of the left renal artery (A1*)	76, 8	116.3	93.8	20.3	21.7
Aortic-mesenteric angle (A2)	64, 5	87.2	74.8	11.5	15.4
Angle of the external iliac arteries (A3)	31, 7	44.0	36.6	6.5	17.8
Aortic-femoral length (L1)	12, 4	13.5	12.9	0.5	4.0
Femoral-renal length (L2)	19, 9	23.6	22.1	1.9	8.6
Femoral-celiac length (L3)	27, 0	31.6	30.0	2.6	8.6
Femoral-subclavian length (L4)	52, 9	62.0	58.9	5.2	8.9

SD = standard deviation; CV = coefficient of variation; number and letters in parenthesis (for example, A1) correspond to the measurement points shown in Figure 1. L1, L2, L3, and L4 represent the lengths from the superficial femoral artery (at the level of the head of the femur) to the following points: terminal aorta, more caudal renal artery, celiac artery, and left subclavian artery, respectively.

### Venous diameters

The external jugulars had much larger diameters than the internal jugulars. The mean diameter of the right external jugular was 9.2 mm and mean diameter of the left external jugular was 8.6 mm. The internal jugular veins had mean diameters of 4 mm on the right side and 4.5 mm on the left side.

The mean diameter of the caudal vena cava, proximal to the level of the more caudal renal vein, was 10.5 mm.

In view of the narrowing of the left renal vein between the aorta and the cranial mesenteric artery (similar to the Nutcracker syndrome), measurements were taken at the renal hilum (mean diameter: 5.8 mm) and at the point of maximum compression (mean diameter: 2.8 mm).

The mean diameter of the right common iliac vein was 7.2 mm; the mean diameter of the left common iliac vein was 6.9 mm proximal to the confluence with the cava and 2.6 mm at the point of maximum compression between the aorta and the adjacent vertebrae (similar to May-Thurner Syndrome). Diameters of the veins are presented in Table 3.

**Table 3:** Venous diameters.

**Diameter venous**	**Minimum**	**Maximum**	**Mean**	**SD**	**CV%**
Right internal jugular (16a)	3.6	4.3	4.0	0.4	9.2
Left internal jugular (16b)	3.8	5.4	4.5	0.8	17.2
Right external jugular (17a)	8, 0	10.5	9.2	1.3	13.8
Left external jugular (17b)	7.7	9.2	8.6	0.8	9.5
Caudal vena cava (18)	9.2	11.7	10.5	1.2	11.8
Left renal vein [RH] (19)	5.3	6.4	5.8	0.5	9.4
Left renal vein [PC] (19*)	1.8	3.4	2.8	0.8	29.6
Right common iliac vein (20)	6.5	7.9	7.2	0.7	10.2
Left common iliac vein (21)	6.6	7.4	6.9	0.4	5.7
Left common iliac vein [PC] (21*)	2.3	2.8	2.6	0.3	11.0

SD = standard deviation; CV = coefficient of variation; RH = renal hilum; PC = point of compression; number and letters in parenthesis (for example, 16a) correspond to the measurement points shown in Figure 1.

### Coefficients of variation

The greatest variations (> 20%) were detected at the following points: left renal vein at the point of maximum compression (CV = 29.6%), internal iliac trunk (CV = 28.4%), right subclavian artery (CV = 23.5%), right external iliac artery (CV = 22.0%), and emerging angle of the left renal artery (CV = 21.7%).

**Figure 2:**
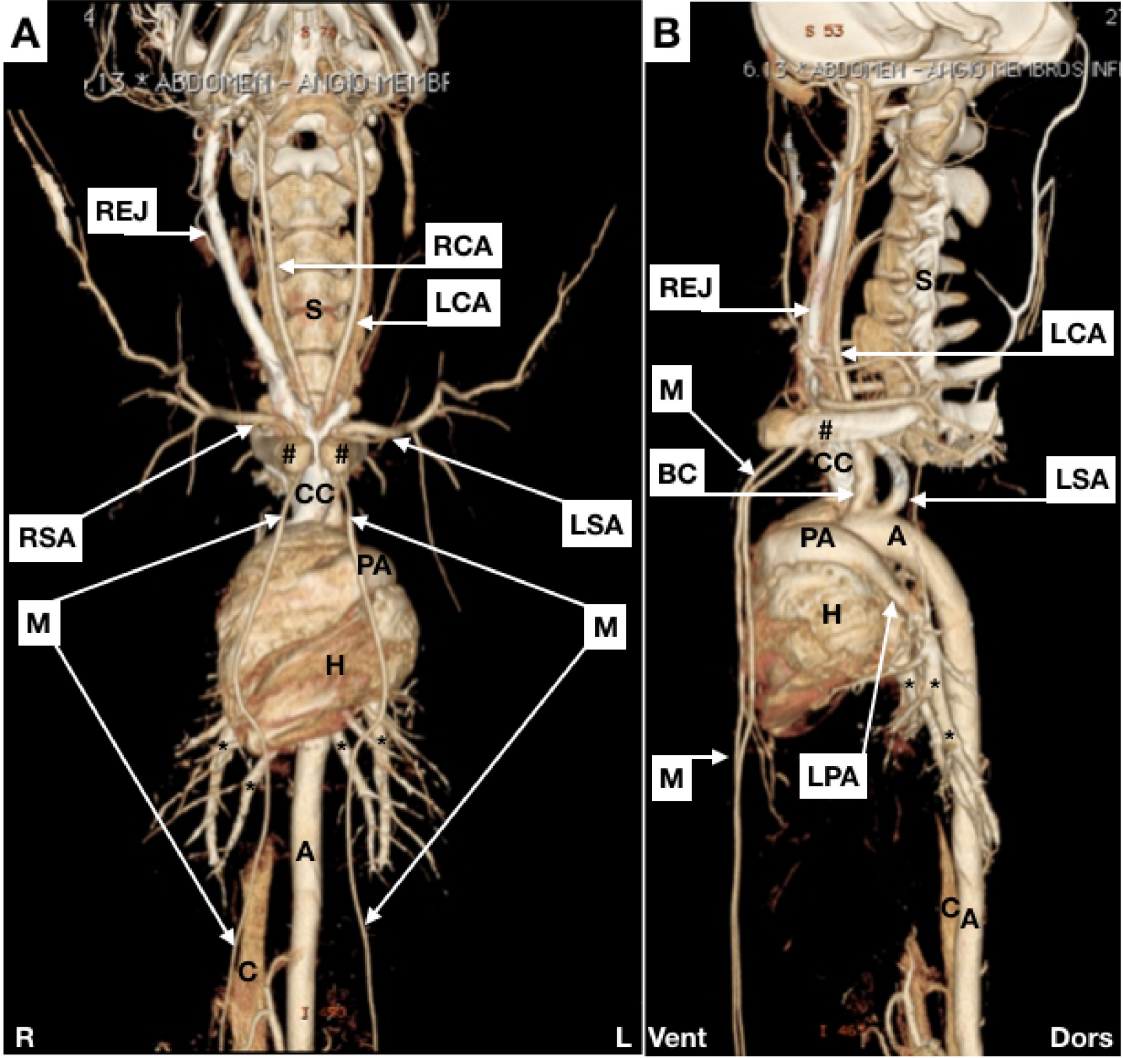
Maximal Intensity Projection (MIP) images. (A) anterior view; (B) lateral view. REJ = right external jugular vein; RCA = right common carotid; LCA = left common carotid; S = spinal column; # = clavicles; CC = cranial vena cava; RSA = right subclavian artery; LSA = left subclavian artery; M = mammary arteries; PA = pulmonary artery (trunk); H = heart; A = aorta; C = caudal vena cava; R = right; L = left; BC = brachiocephalic artery; LPA = left pulmonary artery; * = branches of the pulmonary arteries; Vent = ventral; Dors = dorsal.

Figures 2 through 6 illustrate some of the anatomic information related to arterial and venous anatomy.

**Figure 6:**
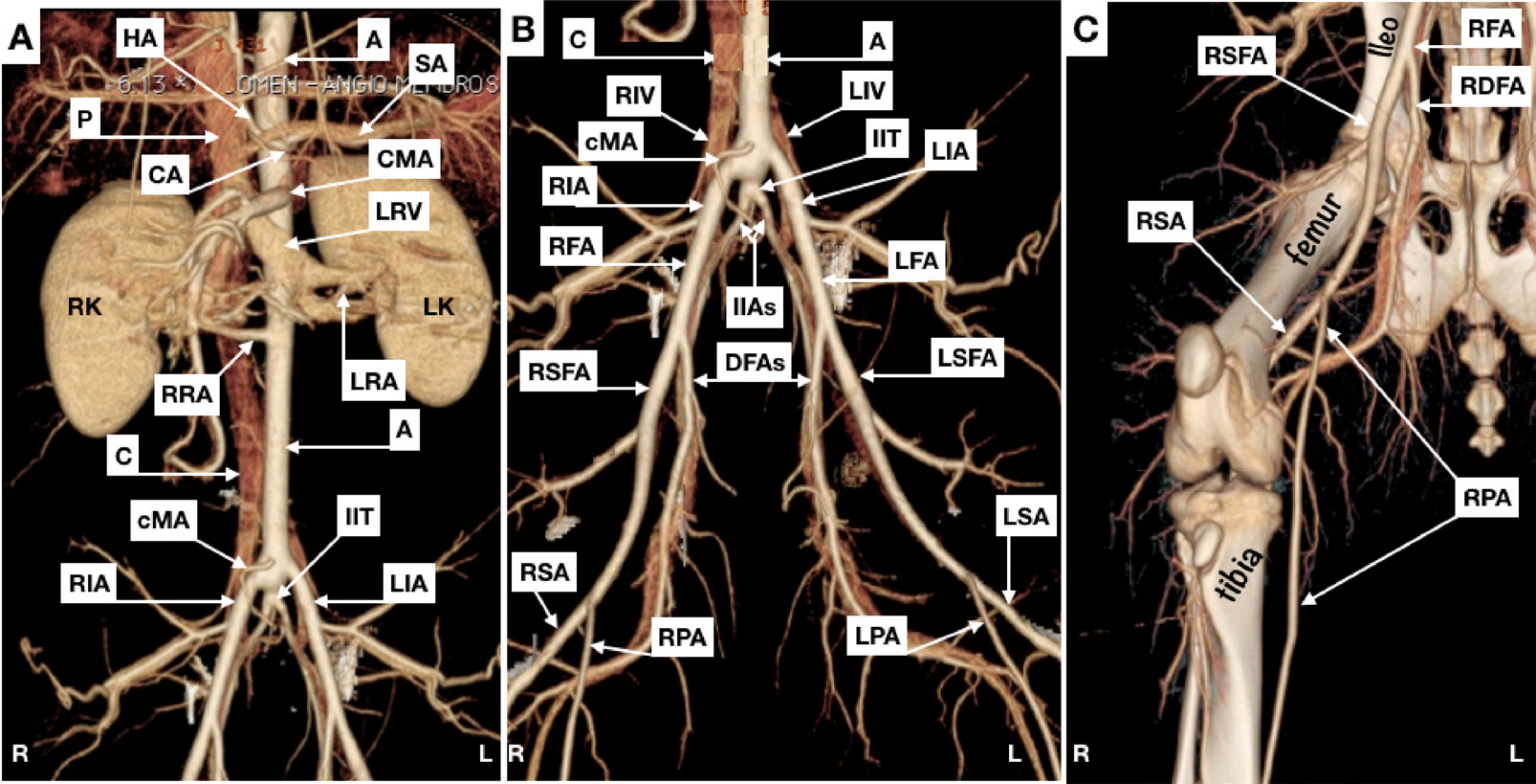
Maximal intensity projection (MIP). (A) abdomen (anterior view); (B) pelvis (anterior view); (C) right hind limb (anterior view). HA = hepatic artery; A = aorta; SA = splenic artery (partially superimposed by the splenic vein); P = portal vein; CA = celiac artery; CMA = cranial mesenteric artery; LRV = left renal vein; RK = right kidney; LK = left kidney; RRA = right renal artery; LRA = left renal artery; C = caudal vena cava; cMA = caudal mesenteric artery; IIT = internal iliac trunk; RIA = right external iliac artery; LIA = left external iliac artery; R = right; L = left; RIV = right common iliac vein; LIV = left common iliac vein; RFA = right common femoral artery; LFA = left common femoral artery; IIAs = internal iliac arteries; RSFA = right superficial femoral artery; DFAs = deep femoral arteries (partially superimposed by the deep femoral vein); LSFA = left superficial femoral artery; RSA = right saphenous artery; RPA = right popliteal artery; LPA = left popliteal artery; LSA = left saphenous artery; RDFA = right deep femoral artery.

### Swine vs. human anatomy

The porcine aortic arch gives rise to two branches: the brachiocephalic and left subclavian arteries; in humans, this structure usually gives rise to the three supra-aortic branches. The brachiocephalic artery gives rise to the right subclavian artery and the bicarotid trunk, which leads to the common carotids (Figure 2).

Another difference is observed at the carotid bifurcation. In humans, the common carotid artery forms a bulb, branching into the internal and external carotids. In pigs, the external carotid artery emerges as a continuation of the common carotid and has a larger caliber than the internal carotid.

Although the study objective did not include describing anatomy in the cephalic region, certain differences compared with human circulation stand out, such as multiple extracranial communications between the external and internal carotid arteries and the *rete mirabile*, a complex ovoid epidural arteriolar network.^2^^,^ 6 ^,^ 9 ^,^ 18

**Figure 3:**
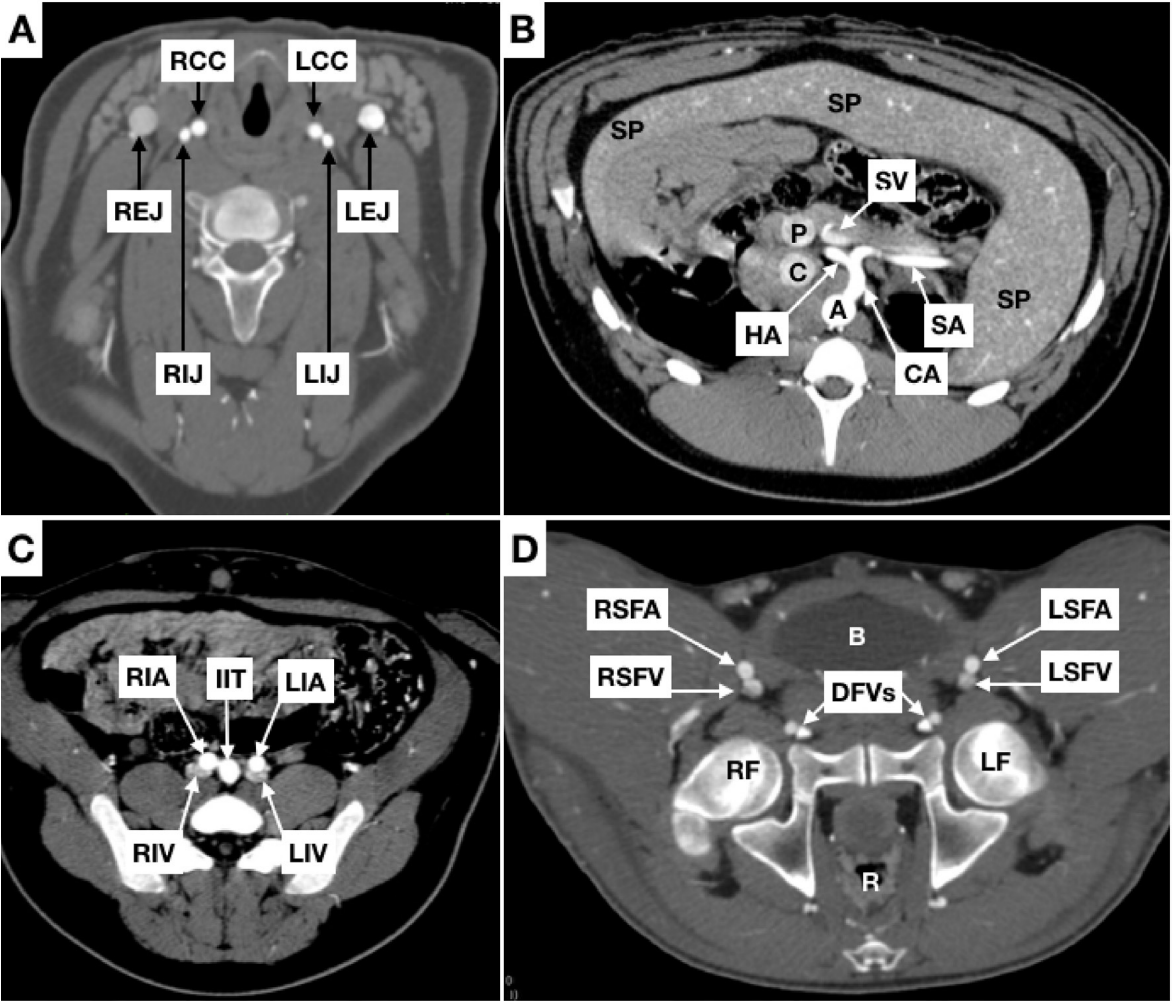
Coronal slices. (A) neck; (B) abdomen; (C) and (D) pelvis. RCC = right common carotid; LCC = left common carotid; REJ = right external jugular vein; LEJ = left external jugular vein; RIJ = right internal jugular vein; LIJ = left internal jugular vein; A = aorta; C = caudal vena cava; P = portal vein; CA = celiac artery; SA = splenic artery; HA = hepatic artery; SV = splenic vein; SP = spleen; RIA = right external iliac artery; LIA = left external iliac artery; RIV = right common iliac vein; LIV = left common iliac vein; IIT = internal iliac trunk; RSFA = right superficial femoral artery; LSFA = left superficial femoral artery; RSFV = right superficial femoral vein; LSFV = left superficial femoral vein; DFVS = deep femoral vessels (arteries and veins); B = bladder; R = rectum; RF = right femur; LF = left femur.

With regard to venous peculiarities, divergent from humans, the external jugular is almost as deep as the internal, but is located within a different compartment. It is also of much larger caliber than the internal jugular, which runs parallel to the carotid (Figure 3A). Additionally, the internal and external jugulars on each side join to the subclavian vein, forming a short trunk which joins to the contralateral equivalent, giving rise to the cranial cava.

**Figure 4:**
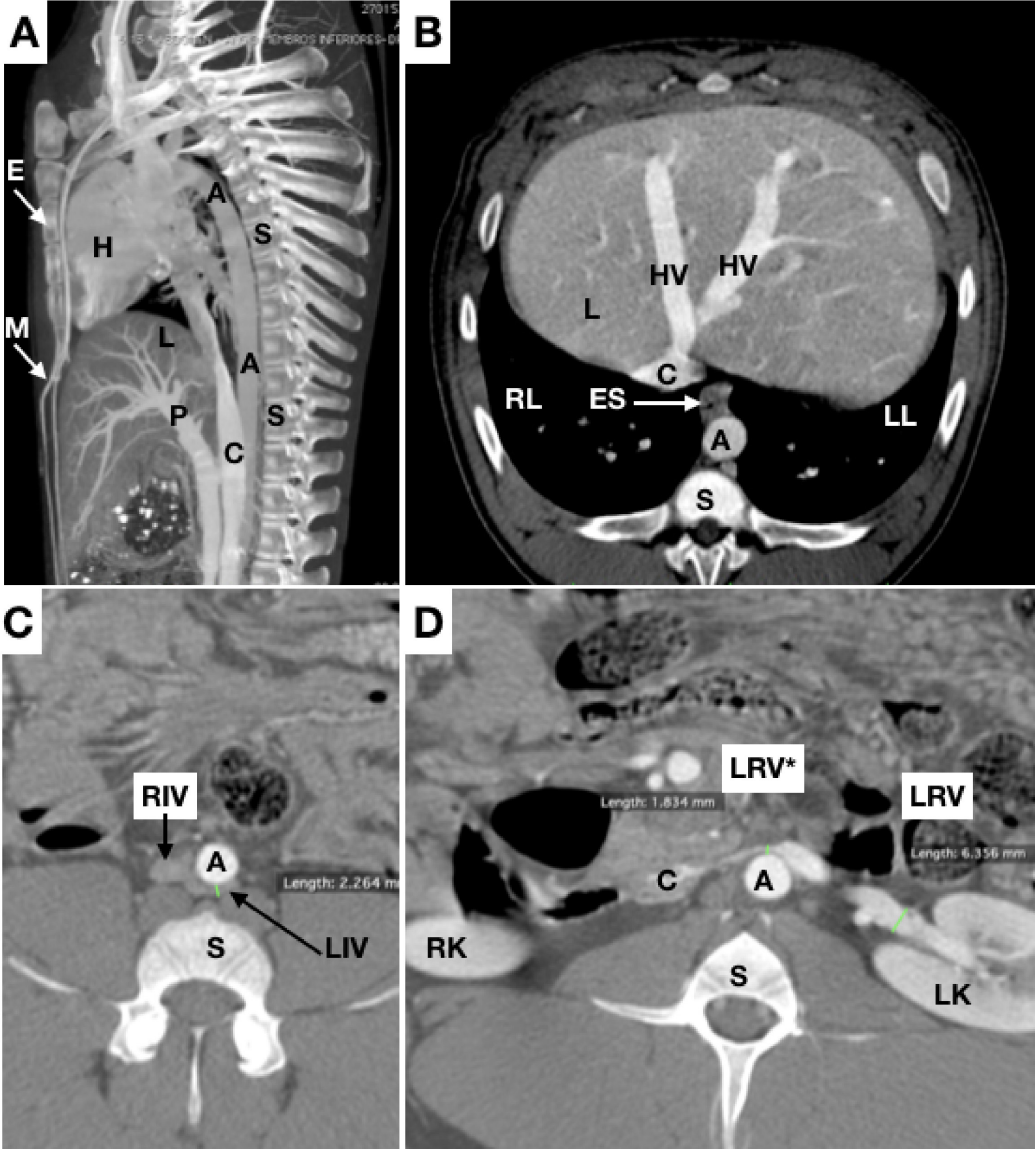
(A) sagittal slices; (B), (C), and (D) axial slices. A = aorta; C = caudal vena cava; P = portal vein; L = liver; S = spinal column; E = sternum; HV = hepatic vein; M = mammary arteries; H = heart; ES = esophagus; RL = right lung; LL = left lung; LRV = left renal vein; LRV* = length from aorta to cranial mesenteric artery; RIV = right common iliac vein; LIV = common iliac vein; RK = right kidney; LK = left kidney.

The thoracic segment of the caudal cava is much longer than the human equivalent (the thoracic segment of the inferior vena cava) and has a portion surrounded by pulmonary parenchyma. The caudal cava crosses the diaphragm and receives the hepatic veins, similar to the retro-hepatic segment of the human inferior cava (Figure 4B).

The renal veins drain to the caudal vena cava. Pigs have a significant narrowing of the left renal vein between the abdominal aorta and the cranial mesenteric artery, analogous to Nutcracker syndrome in humans^19^^-^ 21 (Figures 44D).

The abdominal aorta gives off the celiac, cranial mesenteric, caudal mesenteric, and right and left renal arteries at different angles (Figures 55D).

**Figure 5:**
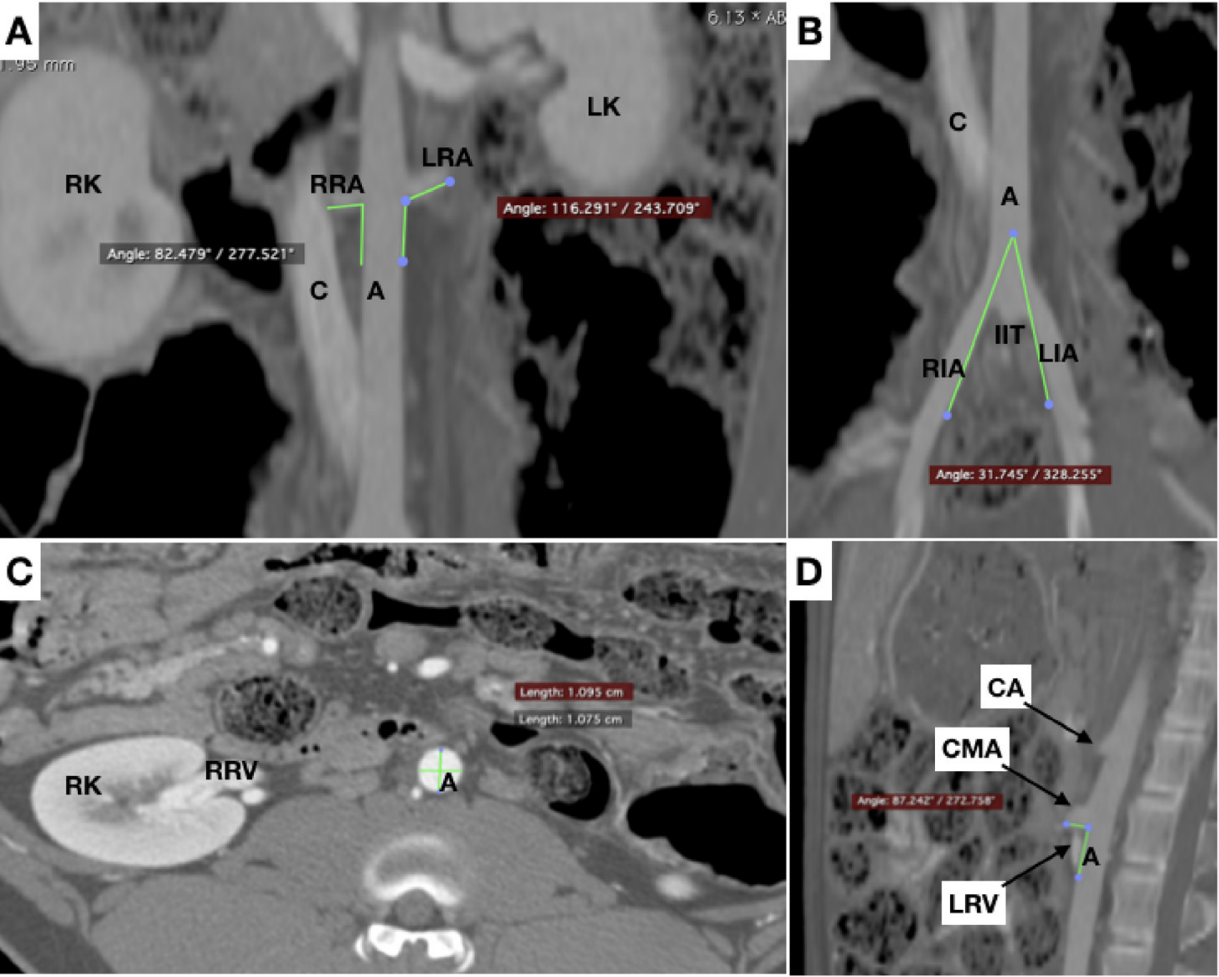
(A) and (B) Coronal slices for measurement of angles between renal arteries and the aorta and between external iliac arteries (trifurcation of the aorta), respectively; (C) Axial slice for measurement of diameter of aorta; (D) Sagittal slice for measurement of the angle between the aorta and the anterior mesenteric artery. A = aorta; C = caudal vena cava; RRA = right renal artery; LRA = left renal artery; RK = right kidney; LK = left kidney; RIA = right external iliac artery; LIA = left external iliac artery; IIT = internal iliac trunk; RRV = right renal vein; CA = celiac artery; CMA = cranial mesenteric artery; LRV = left renal vein.

Pigs do not have a “common” iliac artery, because the aorta ends in a “trifurcation”, giving off right and left external iliac arteries and the internal iliac trunk, which in turn gives rise to the right and left internal iliac arteries and the median sacral artery (Figures 5 B, 66B).

The external iliac artery continues as the femoral artery. In contrast with what is observed in humans, while still within the pelvis, the femoral artery (the equivalent of the human common femoral) branches into superficial and deep femoral arteries; the artery at the prominence of the head of the femur is the superficial femoral, whereas the deep femoral vessels follow a path close to the pubis (Figures 3 D and 6C).

The pelvic venous anatomy is similar to the human: the internal and external iliac veins join to form common iliac veins, which flow together to form the caudal vena cava. We detected compressions of the left common iliac vein, analogous to May-Thurner syndrome; although in pigs the vein narrows between the aorta and the spine, rather than between the right common iliac artery and the spine, as in humans^22^^,^ 23 (Figure 4C).

Arterial supply to the hind limbs diverges considerably from the human equivalent. The superficial femoral artery divides into the saphenous and popliteal arteries at the mid third of the femur. The saphenous artery emits branches that supply the greater part of the musculature, including the plantar and dorsal digital arteries. The popliteal artery penetrates the interosseous membrane in the anterior direction, from where it becomes the cranial tibial artery and supplies several muscular branches.

## DISCUSSION

### Pig models for training and experimentation

Animal experimentation is frequently employed to assess the efficacy and safety of new treatments before they are applied in clinical trials.^1^^-^ 7 ^,^ 9 ^-^ 15 ^,^ 24 Although pigs have been used extensively in many areas, such as genomics, organ transplantation, oncology, trauma, and neurological and cardiovascular diseases,^2^^-^ 4 ^,^ 10 ^,^ 18 many important physiological and anatomic factors remain unknown or have only been described superficially.^1^^,^ 5 ^,^ 25

The literature contains several conflicting anatomic descriptions, such as the “bifurcation” of the aorta,^3^^,^ 6 ^,^ 26 which is actually a “trifurcation” in pigs. Additionally, the subclavian arteries,^3^^,^ 6 ^,^ 11 ^,^ 15 ^,^ 27 which emerge from the brachiocephalic artery on the right side and from the aortic arch on the left side, supplying the animal’s front limbs, are occasionally referred to as brachial arteries.^9^ Another example is the anterior and posterior mesenteric arteries,^9^ which have been described as superior^3^^,^ 11 ^,^ 28 and inferior^3^^,^ 11 ^,^ 15 ^,^ 28 or cranial and caudal.^6^

Several pig breeds have been used in vascular research. Yucatan pigs have been used for studies involving hemodialysis catheters, because this breed stops growing after 1 year of age, with a mean weight of 70 kg, making them appropriate for this use.^13^ At 3 months of age, weighing from 32.4 to 34.5 kg, the same breed has been used to study microsurgical vascular anastomoses in a model of tibia transplantation^9^ and, at 4 months of age, has been used to evaluate differences between the sexes with relationship to the results of coronary stenting.^24^

The Göttingen minipig breed has been used in tomographic studies of vascular anatomy, more specifically with a focus on pediatric issues, since at 11 weeks of life, these animals have approximately the same weight as a newborn human being,^3^ with an appropriate profile for these projects.

The same breed as used in the present study, Landrace, weighing approximately 55 kg were used in experimental construction of aneurysms of the aorta and treatment of types IA and II endoleaks by laparoscopy and thoracoscopy.^13^ At weights from 70 to 95 kg, they were used in a study of resuscitative endovascular balloon occlusion of the aorta (REBOA).^29^ Landrace pigs weighing 20 to 30 kg were also used successfully for training aortic surgery via videolaparoscopy.^26^ In 1998, a radiological anatomy study was conducted with Landrace pigs weighing from 20 to 25 kg, describing angiographic aspects of the brain, head, neck, thorax, abdomen, and pelvis.^6^

In addition to the fact that this breed has already been used for training and research into several open and endovascular surgical techniques, the choice to study Landrace pigs was made because they are widely available and are appropriate for a variety of procedures, depending on the age, size and weight of the animal.

Over time, computed tomography angiography (angio-CT) has supplanted conventional angiography in many situations, because of its many advantages, such as cost and the speed with which the professionals needed to conduct it can be mobilized. It is also less invasive, with lower morbidity and, because of this, it constitutes the method of choice for the circulatory system in many situations.^28^ However, there are few descriptions of the porcine vascular anatomy using tomography.^5^

### Considerations with relation to training and research in vascular procedures

- Vascular access

Vascular access, using the Seldinger technique, can be performed by ultrasound-guided puncture or by surgical dissection.^1^ Dissection of the porcine femoral artery is performed on the superficial femoral artery and not the common femoral artery, since this is intrapelvic in pigs. During dissection of the common carotid, care should be taken to avoid injuring the sympathetic chain, located within the carotid sheath, medial and dorsal to the vagus nerve, since this can lead to Horner syndrome. Local vasodilators may be needed during manipulation of the carotid territory, because spasms occur easily.^1^ As in human beings, the close proximity of the esophagus and left common carotid artery should be kept in mind.

- Coagulation

Pigs are relatively hypercoagulable compared to humans, making intraoperative anticoagulation very important.^1^^,^ 30 As in clinical practice, intravenous heparin (100-300 units/kg) is administered to maintain activated coagulation time ≥ 250 s.^7^ Depending on the procedure (stenting or graft bypass, for example), antithrombotic treatment with acetylsalicylic acid and/or clopidogrel is recommended during the postoperative period.^1^^,^ 11

- Stents

Stent diameters should be suited to the vessel to be stented, with minimal oversizing (1.0 to 1.1 times the diameter of the vessel) to avoid mechanical injuries from excessive dilations, resulting in unreliable safety and efficacy data, while stents that are too small could migrate.^1^ The results presented in Tables 1 and 3 should be of help when choosing stent diameters.

Arteries should be carefully chosen so that stents are implanted in vessels with similar characteristics to those treated in clinical practice. This is particularly important when using drug-eluting stents, since vessels with different histological characteristics may respond differently to the active ingredient released.^1^

- Aortic occlusion

In surgical practice, aortic occlusion is often obtained by clamping or by intraluminal balloon inflation. An experimental study in pigs that involved both histological and biomechanical analyses showed that parietal damage and consequent reduced aortic resistance were more intense when aortic flow was interrupted using clamps than when intraluminal balloon occlusion was used.^31^

The vast collateral circulation explains why the repercussions of acute arterial occlusion can be dramatically different from what is observed in humans. In humans, acute aortic occlusion, in saddle embolism, for example, can cause mortality as high as 30%, whereas in porcine models several oligosymptomatic cases of acute and prolonged occlusion have been observed.^11^

These characteristics suggest that while pig models of acute aortic occlusion are anatomically appropriate for surgical training (such as in the REBOA technique, for example), they can yield results that are imprecise from a physiological perspective.

The REBOA technique was developed as an alternative for thoracotomy to aortic clamping. A complacent balloon is advanced into the aorta and inflated, obstructing the flow to distal circulation, increasing afterload and proximal aortic pressure and improving myocardial and cerebral perfusion, with potentially beneficial effects for patients in deep hemorrhagic shock.^12^^,^ 32 ^,^ 33

Porcine models have been employed for development, training, and studies of REBOA.^12^^,^ 29 ^,^ 32 ^,^ 33 Studies have focused on comparing continuous vs. intermittent^33^^-^ 35 and partial vs. total aortic occlusion,^36^ the ideal duration of occlusion,^37^ and tissue oxygenation,^38^ targeting better results in clinical practice. Our results and the scant published data available suggest that the biological and histochemical repercussions of aortic occlusion in pigs should be judiciously interpreted, since the blood supply to the hind limbs is ensured by anastomoses, such as those between the internal thoracic (mammary) arteries and the epigastric vessels^11^ (Figure 4A illustrates the course of the mammary arteries).

The collateral network of the abdominal wall also includes the deep and lumbar circumflex iliac arteries. Moreover, in proximal occlusions, the proportions of the diameters of the blood vessels involved in perfusion of the hind limbs are much more favorable to immediate collateral perfusion than in humans.^11^ While there is no doubt that porcine models are useful for teaching and practicing these surgical procedures, their peculiarities may mean that the results of occlusion of the infrarenal aorta are not comparable to those obtained in clinical practice.

- Venous procedures

Venous catheters are usually inserted via the external jugular, which has a larger caliber than the internal jugular (Figure 2A), which is the opposite to humans.^6^^,^ 13 ^,^ 16

Pigs have been used in vena cava filters studies.^6^^,^ 39 ^,^ 40 It is possible to puncture the venous femoral axis, but ultrasound-guidance advised, because the vein courses posterior to the artery, as can be observed in Figure 3D, making puncture more difficult.

The way hepatic veins converge to the retro-hepatic segment of the caudal vena cava allows practicing maneuvers used in trauma surgery, such as the Pringle technique and triple vascular exclusion of the liver.

After extensive searches of the literature, no article describing the situations analogous to Nutcracker and May-Thurner syndromes in pigs, as observed in the present study, was identified.

During the bibliographic review, a study describing the development of a novel venous stent employing an ovine animal model was found, which mentioned as a limitation the fact that the stent had been implanted in a vein without stenosis, because of the difficulty of simulating these conditions in animals.^34^ This limitation can be potentially overcome by employing pigs, in which venous compression could be detected prior to the experiment with noninvasive examinations, as demonstrated in the present study.

Since CT examinations were performed with the animals in ventral decubitus and duly hydrated, it is improbable that the narrowing seen in the lef renal and left common iliac veins (Figures 22F) can be attributed by abdominal organs compression, suggesting that there is indeed a mechanism that is more similar to that observed in humans. Future studies could focus on examining these findings of stenosis with intravascular ultrasound, or in developing animal models of venous stenting.

Limitations of the study include the fact that only three animals were studied and that animals of both sexes were not studied, since all three pigs were male.

## CONCLUSIONS

By providing the data presented here, this study should facilitate planning of pre-clinical trials and basic research and improvement of vascular surgery training using porcine models.
