# Supplementary material for: Comparative angiotomographic study of swine vascular anatomy: contributions to research and training models in vascular and endovascular surgery
Source: J Vasc Bras. 2021 May 14;20:e20200086. doi: 10.1590/1677-5449.200086 (PMC8147709; doi:10.1590/1677-5449.200086)
Supplement: Translation

# Translation: Estudo de anatomia comparada suína por angiotomografia: contribuições para modelos de pesquisa e treinamento em cirurgia vascular e endovascular

**Authors:** Adenauer Marinho de Oliveira Góes, Rosa Helena de Figueiredo Chaves, Ismari Perini Furlaneto, Emanuelle de Matos Rodrigues, Flávia Beatriz Araújo de Albuquerque, Jacob Hindrik Antunes Smit, Carolina Pinheiro de Oliveira, Simone de Campos Vieira Abib

**Affiliations:** 1 Centro Universitário do Estado do Pará – CESUPA, Curso de Medicina, Belém, PA, Brasil; 2 Universidade Federal de São Paulo – UNIFESP, Programa de Ciência Cirúrgica Interdisciplinar, São Paulo, SP, Brasil.

**Keywords:** suínos, vasos sanguíneos, procedimentos cirúrgicos vasculares, anatomia comparada, procedimentos endovasculares, angiografia por tomografia computadorizada

## Abstract

**Contexto:**

Modelos com animais de médio e grande porte permitem que pesquisadores avaliem a eficácia e a segurança de procedimentos cardiovasculares em sistemas que se assemelham à anatomia humana e podem ser usados para simular cenários para fins de treinamento. Embora modelos suínos tenham sido extensivamente utilizados, muitos fatores fisiológicos e anatômicos permanecem desconhecidos ou apenas superficialmente descritos.

**Objetivos:**

Descrever a anatomia vascular do suíno por tomografia computadorizada, compará-la à anatomia humana e discutir a aplicação dos modelos porcinos em procedimentos abertos e endovasculares.

**Métodos:**

Três porcos machos da raça *Landrace* foram submetidos a tomografia computadorizada. A anatomia vascular de pescoço, tórax, abdome e membros foi analisada e descrita; foram destacadas similaridades e divergências relevantes entre a anatomia vascular de suínos e de humanos e as implicações em procedimentos vasculares nos suínos.

**Resultados:**

O território carotídeo, o arco aórtico e os ramos terminais da aorta em suínos apresentaram diferenças marcantes quando comparados aos de humanos. Foram detectadas compressões de veias renal e ilíaca comum, ambas à esquerda, semelhantes às encontradas nas síndromes humanas de Nutcracker e May-Thurner. Medidas vasculares (diâmetro, comprimento e ângulos) de diferentes topografias de suínos foram fornecidas.

**Conclusões:**

Os dados fornecidos podem ser úteis para o planejamento de ensaios pré-clínicos e pesquisa básica, bem como para o refinamento do treinamento cirúrgico usando modelos suínos no campo da cirurgia vascular.

## INTRODUÇÃO

Doenças cardiovasculares estão entre os principais problemas de saúde e representam tema primordial de pesquisa biomédica^1^. Alguns estudos preliminares foram realizados em animais de menor porte (camundongos, ratos e coelhos), por apresentarem boa relação custo-benefício^2^. Primatas não humanos (por exemplo, babuínos) também são utilizados quando há necessidade de testes com dispositivos maiores, mas o custo e a complexidade das questões éticas são fatores limitantes. Modelos ovinos e suínos representam uma importante opção para pesquisas na área da cirurgia vascular^2^^-^ 4. Esses animais permitem aos pesquisadores a avaliação da eficácia e da segurança de procedimentos cardiovasculares em sistemas com anatomia semelhante à humana e que podem ser usados em treinamento^1^.

Há muito tempo suínos são utilizados como modelo cirúrgico, particularmente porque apresentam um preço razoável e sua anatomia e fisiologia cardiovasculares são semelhantes às dos seres humanos^1^^,^ 3 ^-^ 8. A anatomia e fisiologia cardíaca suínas foram mais estudadas, como demonstram os estudos sobre distribuição de artérias coronárias, função ventricular, metabolismo cardíaco, eletrofisiologia e desenvolvimento de circulação colateral após infarto agudo do miocárdio^1^.

A recente publicação do genoma e a possibilidade de modificação genética dos suínos aumentaram ainda mais a importância do modelo porcino em pesquisa médica^4^. Um fator chave para o desenvolvimento de modelos experimentais e de treinamento é o conhecimento da anatomia e suas variações^5^. Trabalhos clássicos, baseados em dissecções cirúrgicas, como *The anatomy of domestic animals,* foram publicados em 1910^9^, seguidos por outros que descreveram o uso de suínos para uma variedade de procedimentos^1^^,^ 2 ^,^ 5 ^-^ 8 ^,^ 10 ^-^ 16.

Nas últimas décadas, houve aumento na complexidade das cirurgias abertas e endovasculares. Métodos de imagem não invasivos, como tomografia computadorizada (TC), tornaram-se rotineiros no planejamento terapêutico e no acompanhamento de casos, e estão progressivamente substituindo a angiografia tradicional. Apesar dos avanços técnicos, há carência de descrições detalhadas da anatomia vascular extracardíaca de suínos^3^^,^ 5 ^,^ 6 ^,^ 13.

O objetivo deste estudo foi descrever as características da anatomia vascular de suínos por TC, compará-la à anatomia humana e discutir a aplicação de modelos porcinos para procedimentos cirúrgicos abertos e endovasculares.

## MÉTODOS

Este estudo foi aprovado pelo comitê institucional de ética em experimentação animal.

Três porcos machos da raça *Landrace* foram utilizados para a aquisição de imagens de TC. Seus pesos eram 45,4 kg, 49,2 kg e 52,3 kg. Os animais foram mantidos em alojamento padrão (controle de temperatura e umidade) e permaneceram em jejum 12 horas antes dos exames.

### Protocolo anestésico

O procedimento foi realizado sob anestesia geral e monitoramento por médico veterinário. Para pré-medicação, foram utilizadas injeção intramuscular de cetamina (15 mg/kg) e xilazina (1,5 mg/kg).

Indução anestésica se deu por injeção intravenosa de propofol (2,5-5 mg/kg); a manutenção foi realizada com infusão contínua do mesmo medicamento (0,1-0,2 mg/kg/min). Os animais foram posicionados em decúbito ventral durante a aquisição das imagens. Não houve necessidade de intubação.

### Tomografia computadorizada

As imagens foram obtidas em um tomógrafo de 64 canais, com cortes de 0,625 mm. O contraste iodado endovenoso utilizado foi o loexol (120 mL em velocidade de fluxo de 5 mL/s). As medidas vasculares foram obtidas na fase arterial pela técnica de *bolus-tracking*, e a fase venosa foi iniciada imediatamente após a arterial.

Após a realização da TC, os animais foram utilizados no ensino de habilidades cirúrgicas na instituição e, em seguida, submetidos a eutanásia por injeção intravenosa de cloreto de potássio.

### Análise das imagens

Utilizou-se a plataforma Horos™ v3.1.0 (Horosproject.org). Para descrições anatômicas, os termos “ventral” e “dorsal” foram adotados como correspondentes a “anterior” e “posterior” em humanos. Da mesma forma, “cranial” e “caudal” foram utilizados como equivalentes a “superior” e “inferior”^17^.

Os maiores diâmetros dos vasos foram medidos em dois eixos perpendiculares, obtendo-se a seguir o diâmetro médio naquela topografia.

As medidas foram feitas em marcos anatômicos relacionados a procedimentos cirúrgicos e endovasculares, como angioplastias viscerais, implante de filtro de veia cava e endopróteses de aorta (Figura 1).

**Figura 1:** Ilustração dos pontos de referência para as medidas. *: ponto de aferição do diâmetro venoso no ponto de compressão.

### Análise estatística

Utilizou-se o software *Prism* 6 (*Graphpad Software*, La Jolla, EUA). Foram determinadas média, mediana, desvio padrão e coeficiente de variação (CV).

## RESULTADOS

### Anatomia arterial

Em relação às carótidas, as externas apresentaram diâmetro maior que as internas.

A aorta apresentou estreitamento acentuado e progressivo desde sua origem até sua porção distal, com diâmetro médio de 25 mm na aorta ascendente e de 9,8 mm na aorta terminal.

Entre os ramos viscerais, a artéria mesentérica cranial apresentou maior diâmetro, seguida pela artéria celíaca; a mesentérica caudal teve o menor diâmetro. Os diâmetros arteriais são mostrados na Tabela 1.

**Tabela 1:** Diâmetros arteriais.

**Diâmetro arterial**	**Mínimo**	**Máximo**	**Média**	**DP**	**CV%**
Aorta ascendente (1a)	22,9	28,2	25,0	2,8	11,1
Arco aórtico (1b)	18,7	24,1	21,5	2,7	12,5
Aorta descendente (1c)	16,4	22,5	19,4	3,0	15,6
Aorta a (2a)	12,1	13,4	12,9	0,7	5,4
Aorta abdominal (2b)	8,7	11,1	10,2	1,3	12,6
Aorta abdominal (2c)	9,1	10,8	9,8	0,9	9,1
Tronco braquiocefálico (3)	10,6	13,0	11,5	1,3	11,3
Artéria subclávia direita (4a)	4,3	6,8	5,8	1,4	23,5
Artéria subclávia esquerda (4b)	4,4	6,2	5,6	1,1	19,1
Carótida comum direita (5a)	4,0	5,6	4,8	0,8	17,0
Carótida comum esquerda (5b)	4,7	5,4	4,9	0,4	7,8
Carótida interna direita (6a)	3,5	4,0	3,8	0,3	7,7
Carótida interna esquerda (6b)	3,4	3,7	3,5	0,2	5,0
Carótida externa direita (7a)	4,5	5,5	5,0	0,5	10,6
Carótida externa esquerda (7b)	5,1	5,8	5,4	0,4	6,8
Artéria renal direita (8a)	3,5	5,1	4,4	0,8	18,9
Artéria renal esquerda (8b)	5,1	5,7	5,3	0,4	6,7
Tronco celíaco (9)	4,8	7,1	6,2	1,2	19,6
Artéria mesentérica cranial (10)	6,9	8,2	7,4	0,7	9,8
Artéria mesentérica caudal (11)	2,0	2,6	2,4	0,3	13,6
Artéria ilíaca externa direita (12a)	5,4	8,2	7,2	1,6	22,0
Artéria ilíaca externa esquerda (12b)	6,1	7,3	6,7	0,6	8,6
Tronco ilíaco interno (13)	4,7	8,0	6,1	1,7	28,4
Artéria ilíaca interna direita (14a)	3,7	4,7	4,3	0,6	12,7
Artéria ilíaca interna esquerda (14b)	3,8	4,3	4,1	0,3	6, 4
Artéria femoral superficial direita (15a)	4,9	6,4	5,8	0,8	13,7
Artéria femoral superficial esquerda (15b)	4,8	5,6	5,3	0,4	8,2

DP = desvio padrão; CV = coeficiente de variação; números e letras entre parênteses (por exemplo, 1a) correspondem aos pontos de medidas na Figura 1.

Um animal apresentou artéria renal esquerda mais caudal que a direita; nos outros dois animais, ambas as renais emergiam no mesmo nível da aorta. Os ângulos das artérias renais em relação à aorta diferiram amplamente, de 82,5º a 114,5º (média de 96,8º) à direita e de 76,8º a 116,3º (média de 93,8º) à esquerda.

O comprimento da artéria femoral superficial direita (na altura da cabeça do fêmur) até a aorta terminal (L1) foi em média de 12,9 cm. A partir do mesmo ponto, o comprimento médio foi de 22,1 cm até a artéria renal mais caudal (L2), 30 cm até a artéria celíaca (L3), e 58,9 cm até a artéria subclávia esquerda (L4). Ângulos arteriais e comprimentos estão dispostos na Tabela 2.

**Tabela 2:** Ângulos e comprimentos arteriais.

**Ângulos/comprimento arterial**	**Mínimo**	**Máximo**	**Média**	**DP**	**CV%**
Ângulo da artéria renal direita (A1)	82,5	114,5	96,8	16,3	16,8
Ângulo da artéria renal esquerda (A1*)	76, 8	116,3	93,8	20,3	21,7
Ângulo aórtico-mesentérico (A2)	64, 5	87,2	74,8	11,5	15,4
Ângulo das artérias ilíacas externas (A3)	31, 7	44,0	36,6	6,5	17,8
Comprimento aórtico-femoral (L1)	12, 4	13,5	12,9	0,5	4,0
Comprimento femoral-renal (L2)	19, 9	23,6	22,1	1,9	8,6
Comprimento femoral-celíaca (L3)	27, 0	31,6	30,0	2,6	8,6
Comprimento femoral-subclávia (L4)	52, 9	62,0	58,9	5,2	8,9

DP = desvio padrão; CV = coeficiente de variação; números e letras entre parênteses (por exemplo, A1) correspondem aos pontos de medidas na Figura 1. L1, L2, L3 e L4 representam os comprimentos entre a artéria femoral superficial (na altura da cabeça do fêmur) e os seguintes pontos: aorta terminal, artéria renal mais caudal, artéria celíaca e artéria subclávia esquerda, respectivamente.

### Diâmetros venosos

As jugulares externas apresentaram diâmetro muito maior que as internas. O diâmetro médio da jugular externa direita foi 9,2 mm, enquanto que o da esquerda foi de 8,6 mm; já a jugular interna apresentou diâmetro médio de 4 mm no lado direito e 4,5 mm no lado esquerdo.

O diâmetro médio da veia cava caudal, proximalmente ao nível da veia renal mais caudal, foi de 10,5 mm.

Devido à detecção de estreitamento na veia renal esquerda entre a aorta e a artéria mesentérica cranial (semelhante à síndrome de Nutcracker), as medidas foram feitas no hilo renal (diâmetro médio de 5,8 mm) e no ponto de compressão máxima (diâmetro médio de 2,8 mm).

O diâmetro médio da veia ilíaca comum direita foi de 7,2 mm; o diâmetro médio da veia ilíaca comum esquerda foi de 6,9 mm proximalmente à confluência com a cava e 2,6 mm no ponto de compressão máxima entre a aorta e as vértebras adjacentes (semelhante à síndrome de May-Thurner). Diâmetros venosos são exibidos na Tabela 3.

**Tabela 3:** Diâmetros venosos.

**Diâmetro venoso**	**Mínimo**	**Máximo**	**Média**	**DP**	**CV%**
Jugular interna direita (16a)	3,6	4,3	4,0	0,4	9,2
Jugular interna esquerda (16b)	3,8	5,4	4,5	0,8	17,2
Jugular externa direita (17a)	8, 0	10,5	9,2	1,3	13,8
Jugular externa esquerda (17b)	7,7	9,2	8,6	0,8	9,5
Veia cava caudal (18)	9,2	11,7	10,5	1,2	11,8
Veia renal esquerda [H] (19)	5,3	6,4	5,8	0,5	9,4
Veia renal esquerda [CP] (19*)	1,8	3,4	2,8	0,8	29,6
Veia ilíaca comum direita (20)	6,5	7,9	7,2	0,7	10,2
Veia ilíaca comum esquerda (21)	6,6	7,4	6,9	0,4	5,7
Veia ilíaca comum esquerda [CP] (21*)	2,3	2,8	2,6	0,3	11,0

DP = desvio padrão; CV = coeficiente de variação; H = hilo renal; CP = ponto de compressão; números e letras entre parênteses (por exemplo, 16a) correspondem aos pontos medidos na Figura 1.

### Coeficientes de variação

As maiores variações (> 20%) foram detectadas nos seguintes pontos: veia renal esquerda no ponto de compressão máxima (CV = 29,6%), tronco ilíaco interno (CV = 28,4%), artéria subclávia direita (CV = 23,5%), artéria ilíaca externa direita (CV = 22,0%) e ângulo de emergência da artéria renal esquerda (CV = 21,7%).

Algumas informações anatômicas relacionadas à anatomia arterial e venosa são ilustradas nas Figuras 2 a 6.

**Figura 2:** Imagens em projeção de intensidade máxima (*maximal intensity projection*, MIP). (A) visão frontal; (B) visão lateral. REJ = veia jugular externa direita; RCA = carótida comum direita; LCA = carótida comum esquerda; S = coluna vertebral; # = clavículas; CC = veia cava cranial; RSA = artéria subclávia direita; LSA = artéria subclávia esquerda; M = artérias mamárias; PA = artéria pulmonar (tronco); H = coração; A = aorta; C = veia cava caudal; R = direita; L = esquerda; BC = artéria braquiocefálica; LPA = artéria pulmonar esquerda; * = ramos das artérias pulmonares; Vent = ventral; Dors = dorsal.

**Figura 6:** Imagens em projeção de intensidade máxima (*maximal intensity projection*, MIP). (A) abdome (visão frontal); (B) pelve (visão frontal); C) membro direito posterior (visão frontal). HA = artéria hepática; A = aorta; SA = artéria esplênica (parcialmente sobreposta pela veia esplênica); P = veia porta; CA = artéria celíaca; CMA = artéria mesentérica cranial; LRV = veia renal esquerda; RK = rim direito; LK = rim esquerdo; RRA = artéria renal direita; LRA = artéria renal esquerda; C = veia cava caudal; cMA = artéria mesentérica caudal; IIT = tronco ilíaco interno; RIA = artéria ilíaca externa direita; LIA = artéria ilíaca externa esquerda; R = direita; L = esquerda; RIV = veia ilíaca comum direita; LIV = veia ilíaca comum esquerda; RFA = artéria femoral comum direita; LFA = artéria femoral comum esquerda; IIAs = artérias ilíacas internas; RSFA = artéria femoral superficial direita; DFAs = artérias femorais profundas (parcialmente sobrepostas pela veia femoral profunda); LSFA = artéria femoral superficial esquerda; RSA = artéria safena direita; RPA = artéria poplítea direita; LPA = artéria poplítea esquerda; LSA = artéria safena esquerda; RDFA = artéria femoral profunda direita.

### Anatomia suína × humana

O arco aórtico porcino dá origem a dois ramos: artérias braquiocefálica e subclávia esquerda; em humanos, essa estrutura origina, normalmente, os três ramos supra-aórticos. A partir da artéria braquiocefálica, emergem a artéria subclávia direita e o tronco bicarotídeo, que origina as carótidas comuns (Figura 2).

Outra disparidade ocorre na bifurcação carotídea. Em humanos, a artéria carótida comum apresenta um bulbo e bifurca-se em carótidas interna e externa. Já em suínos, a artéria carótida externa emerge como continuidade da carótida comum e é mais calibrosa que a interna.

Apesar de não fazer parte do objetivo desta pesquisa descrever a anatomia da região cefálica, destacam-se diferenças em comparação à circulação humana, como a presença de múltiplas comunicações extracranianas entre as artérias carótidas externa e interna e da *rete mirabile*, uma complexa rede arteriolar epidural com formato ovoide^2^^,^ 6 ^,^ 9 ^,^ 18.

No que diz respeito às singularidades venosas, divegindo dos humanos, a jugular externa é quase tão profunda quanto a interna, porém está localizada em um compartimento diferente. Também é muito mais calibrosa que a interna, a qual cursa paralela à carótida (Figura 3A). Além disso, as jugulares internas e externas de cada lado se fundem à veia subclávia, formando um tronco curto que, ao se unir ao contralateral equivalente, origina a cava cranial.

**Figura 3:** Cortes coronais. (A) pescoço; (B) abdome; (C) e (D) pelve. RCC = carótida comum direita; LCC = carótida comum esquerda; REJ = veia jugular externa direita; LEJ = veia jugular externa esquerda; RIJ = veia jugular interna direita; LIJ = veia jugular interna esquerda; A = aorta; C = veia cava caudal; P = veia porta; CA = artéria celíaca; SA = artéria esplênica; HA = artéria hepática; SV = veia esplênica; SP = baço; RIA = artéria ilíaca externa direita; LIA = artéria ilíaca externa esquerda; RIV = veia ilíaca comum direita; LIV = veia ilíaca comum esquerda; IIT = tronco ilíaco interno; RSFA = artéria femoral superficial direita; LSFA = artéria femoral superficial esquerda; RSFV = veia femoral superficial direita; LSFV = veia femoral superficial esquerda; DFVs = vasos femorais profundos (artérias e veias); B = bexiga; R = reto; RF = fêmur direito; LF = fêmur esquerdo.

O segmento torácico da cava caudal é muito mais longo que o equivalente no humano (segmento torácico da veia cava inferior) e possui uma porção circundada por parênquima pulmonar. A cava caudal atravessa o diafragma e recebe as veias hepáticas, semelhante ao segmento retro-hepático da cava inferior humana (Figura 4B).

**Figura 4:** (A) corte sagital; (B), (C) e (D) cortes axiais. A = aorta; C = veia cava caudal; P = veia porta; L = fígado; S = coluna vertebral; E = esterno; HV = veia hepática; M = artérias mamárias; H = coração; ES = esôfago; RL = pulmão direito; LL = pulmão esquerdo; LRV = veia renal esquerda; LRV* = comprimido entre a aorta e a artéria mesentérica cranial; RIV = veia ilíaca comum direita; LIV = veia ilíaca comum; RK = rim direito; LK = rim esquerdo.

Na veia cava caudal desembocam as veias renais. Suínos podem apresentar um importante estreitamento na veia renal esquerda, entre a aorta abdominal e a artéria mesentérica cranial, análogo à síndrome de Nutcracker em humanos^19^^-^ 21 (Figuras 44D).

A aorta abdominal emite as artérias celíaca, mesentéricas cranial e caudal, e renais direita e esquerda, em diferentes ângulos (Figuras 55D).

**Figura 5:** (A) e (B) Cortes coronais, aferição dos ângulos entre as artérias renais e a aorta e entre as artérias ilíacas externas (trifurcação da aorta), respectivamente; (C) Corte axial para medir diâmetro aórtico; (D) Corte sagital para medir o ângulo entre a aorta e a artéria mesentérica anterior. A = aorta; C = veia cava caudal; RRA = artéria renal direita; LRA = artéria renal esquerda; RK = rim direito; LK = rim esquerdo; RIA = artéria ilíaca externa direita; LIA = artéria ilíaca externa esquerda; IIT = tronco ilíaco interno; RRV = veia renal direita; CA = artéria celíaca; CMA = artéria mesentérica cranial; LRV = veia renal esquerda.

Suínos não possuem artéria ilíaca “comum”, pois a aorta termina em “trifurcação”, emitindo artérias ilíacas externas direita e esquerda e o tronco ilíaco interno, que por sua vez origina as artérias ilíacas internas direita e esquerda e a artéria sacral mediana (Figuras 5 B, 66B).

A artéria ilíaca externa continua como artéria femoral. De forma divergente ao observado em humanos, a artéria femoral (equivalente à femoral comum em humanos), ainda intrapélvica, ramifica-se em artérias femorais superficial e profunda; a artéria na projeção da cabeça do fêmur é a femoral superficial, enquanto que os vasos femorais profundos percorrem um trajeto próximo ao púbis (Figuras 3 D e 6C).

A anatomia venosa pélvica assemelha-se à humana: as veias ilíacas interna e externa fundem-se em veias ilíacas comuns, que confluem formando a veia cava caudal. Detectamos ocorrência de compressões da veia ilíaca comum esquerda, análogas à síndrome de May-Thuner; contudo, nos suínos o estreitamento venoso ocorre entre a aorta e a coluna vertebral, e não entre a artéria ilíaca comum direita e a coluna vertebral, como em humanos^22^^,^ 23 (Figura 4C).

O suprimento arterial dos membros posteriores diverge bastante daquele equivalente em humanos. A artéria femoral superficial, no terço médio do fêmur, divide-se nas artérias safena e poplítea. A artéria safena emite ramos que suprem grande parte da musculatura, inclusive as artérias digitais plantares e dorsais. A artéria poplítea se anterioriza através da membrana interóssea, passando a ser denominada artéria tibial cranial e fornecendo vários ramos musculares.

## DISCUSSÃO

### Modelos suínos para treinamento e experimentação

Animais de experimentação são frequentemente utilizados para avaliar a eficácia e a segurança de novos tratamentos antes da aplicação em ensaios clínicos^1^^-^ 7 ^,^ 9 ^-^ 15 ^,^ 24. Embora suínos tenham sido extensivamente utilizados em várias áreas, como genômica, transplante de órgãos, oncologia, trauma, doenças neurológicas e cardiovasculares^2^^-^ 4 ^,^ 10 ^,^ 18, muitos fatores fisiológicos e anatômicos importantes continuam desconhecidos ou superficialmente descritos^1^^,^ 5 ^,^ 25.

A literatura apresenta várias descrições anatômicas discordantes, como a da “bifurcação” da aorta^3^^,^ 6 ^,^ 26, que em suínos é, na verdade, uma “trifurcação”. Além disso, as artérias subclávias^3^^,^ 6 ^,^ 11 ^,^ 15 ^,^ 27, que surgem da artéria braquiocefálica no lado direito e do arco aórtico no lado esquerdo, suprindo os membros anteriores do animal, são ocasionalmente chamadas de artérias braquiais^9^; outro exemplo são as artérias mesentéricas anterior e posterior^9^, para as quais também se encontram descrições como superior^3^^,^ 11 ^,^ 28 e inferior^3^^,^ 11 ^,^ 15 ^,^ 28 ou cranial e caudal^6^.

Diferentes raças têm sido usadas em pesquisa vascular. Porcos *Yucatan* foram utilizados para estudos envolvendo cateteres de hemodiálise, pois essa raça para de crescer após 1 ano de idade, com peso médio de 70 kg, tornando-os apropriados para esse propósito^13^. Essa mesma raça, aos 3 meses de idade, com peso entre 32,4 e 34,5 kg, foi utilizada para anastomoses vasculares microcirúrgicas em um modelo de transplante de tíbia^9^; e, com 4 meses de idade, foi usada para avaliar a diferença entre os gêneros, em relação aos resultados de stents coronarianos^24^.

A raça de miniporco *Göttingen* foi utilizada em estudos de anatomia vascular por tomografia, mais especificamente com enfoque em problemas pediátricos, visto que, com 11 semanas de vida, esses animais têm o peso aproximado do ser humano ao nascer^3^, com um perfil adequado para esses projetos.

Porcos da raça *Landrace*, a mesma utilizada no presente estudo, pesando aproximadamente 55 kg, foram utilizados no desenvolvimento experimental de aneurismas de aorta e no tratamento de *endoleaks* tipos IA e II por laparoscopia e toracoscopia^13^. Com peso entre 70 e 95 kg, foram usados para pesquisa com oclusão ressuscitativa por balão endovascular da aorta (*resuscitative endovascular balloon occlusion of the aorta*, REBOA)^29^. Suínos *Landrace* com peso entre 20 e 30 kg também foram utilizados com sucesso para o treinamento de cirurgia aórtica por videolaparoscopia^26^. Em 1998, um estudo de anatomia radiológica feito em porcos *Landrace* pesando entre 20 e 25 kg descreveu aspectos angiográficos de cérebro, cabeça, pescoço, tórax, abdome e pelve^6^.

A opção por estudar essa raça, além de esta já ter sido utilizada em treinamentos e pesquisas de várias técnicas cirúrgicas abertas e endovasculares, se deve ao fato de a raça estar facilmente disponível e ser adequada a uma variedade de procedimentos, dependendo da idade, do tamanho e do peso do animal.

Com o tempo, a angiotomografia computadorizada (angio-TC) superou a angiografia convencional em muitos cenários, devido às suas diversas vantagens, como a economia e a rapidez para mobilizar profissionais necessários para execução. Também é menos invasiva e mórbida e, por isso, representa a modalidade de escolha para o sistema circulatório em diversas situações^28^. No entanto, descrições da anatomia vascular suína por tomografia são limitadas^5^.

### Considerações sobre modelos de treinamento e pesquisa em procedimentos vasculares

- Acesso vascular

O acesso vascular, utilizando a técnica de Seldinger, pode ser realizado por punção guiada por ultrassonografia ou dissecção cirúrgica^1^. A dissecção da artéria femoral é realizada na artéria femoral superficial e não na femoral comum, devido ao fato de esta ser intrapélvica nos suínos. Durante a dissecção da carótida comum, devem-se evitar lesões da cadeia simpática, localizada dentro da bainha carotídea, medial e dorsal ao nervo vago, o que pode resultar em síndrome de Horner. Vasodilatadores locais podem ser necessários durante a manipulação do território carotídeo, pois o espasmo ocorre com facilidade^1^. Assim como nos seres humanos, a relação próxima entre o esôfago e a artéria carótida comum esquerda deve ser lembrada.

- Coagulação

Comparados aos humanos, suínos apresentam hipercoagulabilidade, tornando a anticoagulação intraoperatória muito importante^1^^,^ 30. Como na prática clínica, administra-se heparina intravenosa (100-300 unidades/kg), mantendo o tempo de coagulação ativado ≥ 250 s^7^. Dependendo do procedimento (implante de stent, *bypass* com próteses, por exemplo), complementa-se a terapia antitrombótica com ácido acetilsalicílico e/ou clopidogrel no período pós-operatório^1^^,^ 11.

- Stents

O diâmetro deve ser apropriado ao vaso no qual serão implantados, com sobredimensionamento mínimo (1,0 a 1,1 vezes o diâmetro do vaso), para evitar que lesões mecânicas por dilatação excessiva gerem dados não fidedignos sobre segurança e eficácia, enquanto que stents muito pequenos podem migrar^1^. Resultados apresentados nas Tabelas 1 e 3 podem auxiliar na escolha do diâmetro dos stents.

As artérias devem ser cuidadosamente selecionadas, para que os stents sejam implantados em vasos com características semelhantes às dos tratados na prática clínica. Isso é particularmente importante ao usar stents farmacológicos, pois vasos com características histológicas distintas podem responder de forma variada ao princípio ativo liberado^1^.

- Oclusão aórtica

Na prática cirúrgica, a oclusão aórtica frequentemente é feita pela aplicação de pinças ou pela insuflação intraluminal de balões. Um estudo experimental em suínos, o qual incluiu análises histológicas e biomecânicas, revelou que os danos parietais e consequente diminuição da resistência aórtica são mais intensos quando a interrupção do fluxo aórtico é realizada com a aplicação de pinças do que quando é utilizada a oclusão intraluminal por balão^31^.

A circulação colateral exuberante explica porque a repercussão da oclusão arterial aguda pode ser dramaticamente diferente daquela observada em humanos. Em humanos, a oclusão aórtica aguda, em uma embolia a “cavaleiro”, por exemplo, pode atingir 30% de mortalidade, enquanto que, em modelos suínos, foram encontrados vários casos oligossintomáticos de oclusão aguda e prolongada^11^.

Essas características sugerem que modelos suínos de oclusão aórtica aguda, embora anatomicamente adequados para o treinamento cirúrgico (como a REBOA, por exemplo), podem fornecer resultados imprecisos quanto à perspectiva fisiológica.

A técnica de REBOA evoluiu como alternativa ao clampeamento aórtico por toracotomia. Um balão complacente é introduzido na aorta e insuflado, obstruindo o fluxo para a circulação distal, aumentando a pós-carga e a pressão aórtica proximal, melhorando as perfusões miocárdica e cerebral com efeitos potencialmente benéficos em pacientes com choque hemorrágico profundo^12^^,^ 32 ^,^ 33.

Modelos suínos têm sido utilizados para o desenvolvimento, treinamento e pesquisas sobre REBOA^12^^,^ 29 ^,^ 32 ^,^ 33. As pesquisas enfocam comparações entre oclusão aórtica contínua e intermitente^33^^-^ 35, parcial versus total^36^, tempo ideal de oclusão^37^ e oxigenação tecidual^38^, visando melhores resultados na prática clínica. Nossos resultados e os escassos dados da literatura sugerem que repercussões biológicas e histoquímicas da oclusão aórtica nos suínos devem ser criteriosamente interpretadas, pois o suprimento sanguíneo dos membros posteriores é assegurado por anastomoses, como as que existem entre as artérias torácica interna (mamária) e os vasos epigástricos^11^ (a Figura 4A demonstra o curso das artérias mamárias).

A rede colateral da parede abdominal inclui ainda as artérias ilíacas circunflexa profunda e lombares. Além disso, em caso de oclusão proximal, as proporções de diâmetro dos vasos sanguíneos envolvidos na perfusão dos membros posteriores favorecem muito mais a imediata perfusão colateral do que em humanos^11^. Embora não haja dúvida de que os modelos suínos sejam úteis para ensinar e praticar tais procedimentos cirúrgicos, essas particularidades podem tornar os resultados da oclusão da aorta infrarrenal não comparáveis aos obtidos na prática clínica.

- Procedimentos venosos

Cateteres venosos são usualmente inseridos pela jugular externa, mais calibrosa do que a interna (Figura 2A), uma situação oposta à encontrada em humanos^6^^,^ 13 ^,^ 16.

Suínos têm sido utilizados para estudos sobre filtro de veia cava^6^^,^ 39 ^,^ 40. A punção do eixo femoral é possível; porém, recomenda-se que seja guiada por ultrassom, uma vez que a veia passa posteriormente à artéria, como observado na Figura 3D, dificultando a punção.

O modo como as veias hepáticas convergem para o segmento retro-hepático da veia cava caudal permite o treinamento de manobras usadas em cirurgia do trauma, como a de Pringle e a tríplice exclusão vascular hepática.

Após extensa busca na literatura, não foram encontrados artigos que descrevessem as situações análogas às síndromes de Nutcracker e May-Thurner em suínos, como as relatadas no presente estudo.

Durante a revisão bibliográfica, foi encontrado um estudo sobre o desenvolvimento de um stent venoso utilizando modelo animal ovino; entre as limitações mencionadas, estava o fato de que o stent havia sido implantado em uma veia sem estenose, pela dificuldade de simular tais condições em animais^34^; uma limitação potencialmente superada utilizando-se suínos cuja compressão venosa poderia ser detectada pré-experimento com exames não invasivos, conforme demonstrado no presente estudo.

Como a TC foi realizada com animais em decúbito ventral e devidamente hidratados, é improvável que o estreitamento descrito para as veias renal e ilíaca comum esquerdas (Figuras 22F) seja provocado pelo peso dos órgãos abdominais, sugerindo, de fato, um mecanismo mais semelhante ao encontrado em humanos. Estudos futuros podem se concentrar em examinar esses achados de estenose com ultrassom intravascular, ou em desenvolver modelos animais para implante de stents venosos.

Entre as limitações do estudo, encontram-se o fato de que apenas três animais foram estudados; também não foram estudados animais de ambos os sexos, pois os três animais eram machos.

## CONCLUSÕES

Ao fornecer os dados aqui apresentados, este estudo pode facilitar o planejamento de ensaios pré-clínicos, as pesquisas básicas e o aperfeiçoamento do treinamento cirúrgico utilizando modelos suínos na área vascular.
